# Looking at the ‘field’ through a Zoom lens: Methodological reflections on conducting online research during a global pandemic

**DOI:** 10.1177/1468794120985691

**Published:** 2022-06

**Authors:** Marnie Howlett

**Affiliations:** Department of International Relations, London School of Economics and Political Science, UK

**Keywords:** Methodology, qualitative research, field research, online methods, pandemic

## Abstract

For many social science scholars, the COVID-19 pandemic has forced us to re-think our approaches to research. As a result of new social distancing measures, those of us who conduct in-person qualitative and ethnographic research have faced significant challenges in accessing the populations and fields we study. Technology served as an incredibly useful tool for social interaction and research prior to the pandemic, and it has since become even more important as a way to engage with others. Although not all types of social research, or even all projects, lend themselves to online activities, digital communication platforms like Zoom, Skype, and Facebook have allowed many of us to continue our studies from a distance—in some cases, significant temporal and spatial distances away from our research sites. As such, it is important to consider how these different methodological approaches challenge our understandings of fieldwork. While the disadvantages of not physically accessing the places we study are clear, can mediated approaches offer (any) hope of the immersion we experienced with in-person fieldwork? If many of us are able to continue ethnographic research (in some form) without co-locating with our participants in our field sites, how are our studies fundamentally affected, as well as the ways we conceptualize the ‘field’ more largely? This paper explores these methodological and epistemological questions through reflections on conducting online research during the beginning of the COVID-19 pandemic.

‘[T]he “field” includes: the academy, where research is initiated, where the people we speak with live, and the social contexts and settings in which research is funded and made available to various audiences.’

(Nast, 1994, as cited in Till, 2001: 47)

The COVID-19 pandemic has fundamentally altered the way social science research is conducted. At least temporarily, we are limited in our ability to access the resources we draw on to read, write, analyze, and publish. This includes the materials locked away in libraries and archives, as well as the countries and participants we study; many of which will be inaccessible indefinitely or, at the very least, need to be accessed in new ways. As we are also situated within a wider global context of the pandemic—and the societies and people we study have similarly been affected by the virus—the ways participants engage with us are, and will continue to be, implicated going forward. The recent changes have thence proven particularly difficult for those of us whose projects involve fieldwork. Often understood as research that is ‘based on personal interaction with research [participants] in their own setting’ for an extended period of time (Wood, 2007: 123), field studies involving immersive in-person contact have become nearly impossible for the foreseeable future. Even when a ‘new normal’ is established, many of our previously used approaches to research will likely need to be re-thought and altered, at least temporarily.^1^ As a result, many of us have been, or will be, forced to change our research plans and/or find new techniques to carry out our projects.

In some cases, the only way to continue with our original research plans during the pandemic has been to move away from the physical realm and into a digital reality. Importantly, online research is not a new phenomenon in the social sciences—there are many examples of surveys, content analyses, and digital ethnographies, or ‘netnographies,’ involving existing online material and social media platforms (see Beaulieu, 2004; Bluteau, 2019; Coleman, 2010; De Seta, 2020; Hine, 2000, 2005). Interviews over phone, Skype, and instant messaging software have similarly been used by social science researchers for many years (see Cater, 2011; Deakin and Wakefield, 2014; Jenner and Myers, 2019; Johnson et al., 2019; Sullivan, 2012), and crowdsourcing techniques using the Internet have also proven to be effective in disciplines like psychology and economics. Unlike these earlier projects, though, we now *must* use mediated approaches to avoid in-person interactions, at least if we hope to achieve similar research aims and solve the puzzles we initially proposed.^2^ Moreover, the transition from immersive methods to more ‘hands-off’ approaches occurred quite quickly as national governments rapidly responded to COVID-19 in March 2020 with lockdowns and border closures, yet deadlines for thesis submissions, funding, and publications remained in place. As such, the methods many of us came to employ, or will be employing, were not part of our original research plans nor ones with which we have had much training or experience, or even gave much thought to, prior to the pandemic.

Thus far, the academic community has responded positively to these new challenges, including producing several Twitter threads with relevant materials and a crowdsourced document about doing fieldwork in a pandemic (see Lupton, 2020). In suggesting alternative approaches for data collection, these resources highlight (both in their content and digital nature) that technology can be used for research across different disciplines. For qualitative researchers, and especially ethnographers, mediated approaches resolve some of the limitations caused by social distancing measures and our inability to access the fields and populations we study (Lobe et al., 2020). But while these methods are proving exceptionally helpful for the continuation of many research projects, we must ask if, and how, their use might (re)shape our understandings of ‘fieldwork.’ If we are able to conduct qualitative and ethnographic studies (in some form) from significant temporal and spatial distances away from our participants, how does this fundamentally change our projects and the data we collect? Though the disadvantages of not being able to access our research sites are clear, can mediated approaches offer (any) hope of the immersion we experienced with in-person fieldwork? If so, what might be the role of digital methods, and the ‘field’ more broadly, in social science research in both the short and long term? This paper explores these methodological and epistemological questions through reflections on conducting qualitative interviews and focus groups in-person prior to, and online during, the COVID-19 pandemic.

## Online fieldwork

Fieldwork has been a critical approach to research for many decades. In direct contrast to armchair anthropology—wherein academics base their theories and conclusions on others’ studies without necessarily interacting with the studied populations—fieldwork is a form of inquiry that involves researchers entering a new context, a ‘field’ site (or sites), to carry out their investigation. While some scholars suggest that neither a foreign context nor extensive interpersonal interaction is required, as field research is simply leaving one’s ‘home institution in order to acquire data, information, or insights that significantly inform one’s research’ (Kapiszewski et al., 2015: 1), others emphasize the need to immerse oneself in ‘a collective way of life for the purpose of gaining firsthand knowledge about a major facet of it’ (Shaffir and Stebbins, 1991: 5). For many qualitative studies, especially those more ethnographic in nature, the importance of fieldwork is thus that it allows for immersive engagement with participants ‘in their own setting’ (Wood, 2007: 123). By using in-person techniques like interviews, surveys, and participant observation in various field settings, social science scholars have accordingly been able to answer fundamental questions about the political and social world.

But while field research, and particularly ethnographic field research, has predominantly been understood as immersion in a field site, the Internet has been used across disciplines in recent years to answer similar qualitative questions. Fittingly, a quickly growing body of literature has emerged around new ‘online,’ ‘Internet,’ and ‘computer-mediated’ methods resembling those also used when conducting field research (for example, Coleman, 2010; Fielding et al., 2017; Hine 2015; Pink, 2013; Pink et al., 2016).^3^ Of these online synchronous methods, interviews and focus groups in particular have sparked a discussion because of their widespread uses (Hooley et al., 2012; James and Busher, 2009; Salmons, 2015). Though online real-time interviews were once limited to video platforms, such as Skype, there has recently been a surge in the number of communicative technologies available for conducting these interactions—including social media channels like Facebook, WhatsApp, Viber, and Instagram—which has made it increasingly easier to engage with participants in different settings (Deakin and Wakefield, 2014; Jowett et al., 2011). Markham (2009) posits that these new platforms offer similar opportunities to explore new theories, shed light on undescribed phenomena, and experiment with innovative methodologies in order to study topics including, but not limited to, human behavior and experience.

As an alternative to, or substitute for, conventional face-to-face interviews, mediated approaches like videoconferencing are thus particularly useful for data collection. The real-time nature of the exchanges can resemble the ‘honesty’ of onsite interviews (O’Connor and Madge, 2017), as the dynamic environments prevent participants from overthinking their answers or considering the most socially desirable responses (Mann and Stewart, 2000). Video calling also allows researchers to access verbal and nonverbal cues, providing an equally authentic experience to in-person interviews (Sullivan, 2012). When compared to asynchronous methods or other types of synchronous interviews, such as those conducted over instant messaging, this type of interview ensures a more personable interaction, including greater spontaneity in enabling respondents to answer questions immediately. In the case of focus groups, mediated approaches additionally resemble face-to-face communications in similarly allowing participants to interact with one another (Chen and Hinton, 1999). Due to the significant advancements in technology, in-person interactions are therefore no longer ‘the gold standard against which the performance of computer-mediated interaction is judged’ (Hine, 2005: 4), as online methods are indeed equally valid and legitimate approaches to research.

Nevertheless, Deakin and Wakefield (2014) argue that mediated approaches are still often presented as the ‘second choice’ to the ‘gold standard’ of in-person communication. This is because, at the most practical level, the relative novelty of these newer techniques is a trade-off for more stable tried-and-tested approaches. Abidin and De Seta further purport that online methods often cause ‘anxieties, challenges, concerns, dilemmas, doubts, problems, tensions, and troubles’ (2020: 9) due to perceived difficulties around managing the interactions and generating meaningful conversations. The most obvious discomfort, described from even the earliest attempts at online research (see, for example, Clodius, 1994), is related to the ways new forms of interaction made available by digital media undermine traditional understandings of participation and immersion. In particular, the ‘head shot’ provided by a camera is thought to create an obstacle to fully observing participants’ body language and tone (Cater, 2011), in addition to technical issues like audio and visual clarity. As some disciplines privilege knowledge from immersion in a field site as being thicker and more rigorous research, in-person approaches continue to be perceived as superior and able to generate richer and higher quality data due to the more conversational nature of the interactions (Johnson et al., 2019). The assumed lack of reflection and reflexivity surrounding online communications also appears to challenge the starting premises about ‘who we study, where they are, and what they do there’ (Hine, 2013: 2, also Jowett et al., 2011). Hence, after decades of growing literature surrounding the use and viability of mediated methods, there remains little consensus on their suitability and validity for research, or about whether technologically mediated interactions can adequately replace in-person methods.

But although converging opinions remain, the reality brought about by the COVID-19 pandemic has left many scholars in positions wherein they *must* use mediated approaches to abide by new social distancing measures. While several disciplines have been collecting data online for many years, researchers who conduct qualitative and ethnographic research through fieldwork have found themselves in particularly precarious positions in being forced to replace their immersive in-person interactions with more hands-off approaches. As mediated methods were not part of original research plans, nor ones qualitative scholars necessarily had much training in or experience with due to the fact that methods courses have engaged less with online approaches, many researchers are now both learning and training these techniques for the first time. The adoption of online approaches thus raises new methodological and epistemological concerns around understandings of presence, field relations, observation, and (not) ‘being there’ in one’s field site (Hannerz, 2003: 202). Since a distinct notion of place within a larger ‘field’ is absent when using mediated methods (Beaulieu, 2004), conducting fieldwork without physically co-locating with the people we study additionally begs the question of whether digital methods are appropriate to answer the same research questions without the same immersive experiences.

As the remainder of this paper will thence demonstrate through reflections on qualitative in-person interviews and focus groups conducted in Ukraine between late 2018 and early 2020 and online interactions during the spring of 2020: mediated approaches can be immersive in ways not typically discussed or even previously realized. Although not always beneficial, or even an adequate substitute for all projects, digital methods can support similar ethnographic research by encouraging co-presence with our participants and by helping us embed ourselves in our research sites from afar. While the COVID-19 pandemic has brought much uncertainty for academia, it has evidently also challenged, and will continue to challenge, previously held notions about fieldwork. Accordingly, this article sparks a larger, and very necessary, discussion about how we understand the ‘field’ and the role of mediated methods in qualitative and ethnographic research going forward.

## Fieldwork pre-pandemic

My research employs a mixed method approach using interviews, focus groups, and cognitive mapping exercises to understand how conceptualizations of space, place, and territory shape self-identifications and nationalism in Ukraine. Prior to the COVID-19 pandemic, I spent 16 weeks in three regions of the country conducting 48 ‘elite’ interviews with academics, journalists, politicians, and activists, in addition to 26 focus groups with citizens from diverse backgrounds representing three age groups: 18–29 years, 30–49 years, and 50 years and older.^4^ I also actively engaged in participant observation—mainly, volunteering in various capacities and attending local events, concerts, and festivals with gatekeepers and participants. I had planned my final six-week research trip for April–May 2020, but as Ukraine closed its borders indefinitely on 17 March 2020 in response to the COVID-19 pandemic, I was forced to cancel my visit. As it was uncertain at the time as to how long the country’s borders would be closed, and when my institution would again allow faculty and students to conduct overseas fieldwork, I made the decision in May 2020 to move my remaining data collection online.^5^

Notably, I was not particularly keen to conduct research remotely. This is because I expected the experience to be limiting in both an immersive sense and in terms of the data I would be able to collect—I anticipated my participants would want to speak primarily about COVID-19 and the lockdown they were experiencing in Ukraine. Although the data collected through in-person interviews and focus groups were indubitably different than those collected online amidst the pandemic, the fundamental distinction was not in the content of the conversations, but rather in my observations of my participants and field sites through the use of digital methods. The following pages thus outline my experiences from the first 13 ‘elite’ interviews and one focus group that I conducted online from London, UK during late May and early June 2020. All conversations except for one were video discussions: eleven were held over Facebook video chat and one on each Zoom, Skype, and Viber. It must be stated that the unique situation caused by the COVID-19 pandemic indeed created the backdrop for these interactions, especially because of the specific themes that I study; however, my experiences speak beyond the peculiarities of my project and type of fieldwork to the methodology of online or non-immersed fieldwork for qualitative research more largely.

## Locating the ‘field’ in online fieldwork

### Online co-presence

As ethnographic fieldwork has traditionally been based on ideas about locality and physical immersion in geographically defined research areas (Wittel, 2000), which determine where and how we interact with our participants, online approaches very much complicate the ‘*placeness* of ethnography’ (Haverinen, 2015: 82). When I conducted fieldwork in Ukraine prior to the pandemic, the interviews and focus groups took place in locations wherein my participants and I shared a physical space, such as in offices, cafés, and libraries. For those held exclusively online, conversely, my participants and I were physically separated and located in our respective countries and homes. During these conversations, ten participants sat at tables in rooms by themselves, two sat on couches in their living rooms, and one walked outside. Throughout five of the calls, another person came into the room where the participant was located or was noticeably in their shared space (e.g. I could hear another person or the participant paused the call to chat with someone beyond the camera), and four of my interlocutors changed their location over the course of their interview, such as by moving to another room. Of the five focus group participants, three sat alone on couches or at kitchen tables inside their homes, one sat outside at a table beside his family’s garden in rural Ukraine, and the fifth’s location was unknown as she chose to keep her camera off. Although perhaps seemingly minute details, these settings very much encourage us to reflect on previously held notions about the ‘field’ in fieldwork.

In particular, the different locations of the in-person and online interactions complicate traditional understandings about accessing and entering a ‘field site.’ Rather than traveling to, and fully immersing myself within, the places that I study, my access to Ukraine during the pandemic was determined entirely by my participants’ willingness to invite me into their ‘worlds’ on the ground (Tillmann-Healy, 2003); their decision to answer my call or click a link allowed me to enter, whereas the end of the call equally precluded me from it. The reality of being located in different places was further underscored by the question, ‘where are you right now?,’ which was posed at the beginning of every conversation either by me or my participants. As location is ambiguous in cyberspace, shifting the interaction from offline ‘co-location’ to online ‘co-presence’ (Beaulieu, 2010) through the use of communicative technologies thus aided my participants and I in actively constructing a new digital and socially meaningful space for our interactions that was neither our present locations, nor a common physical setting. Although contrary to the idea of field research as the act of visiting a new setting away from one’s office or institution (Kapiszewski et al., 2015), the use of online methods during the pandemic still very much helped me to ‘ground’ me in a site with my participants even as our bodies were merely staring at screens within our respective homes (Beaulieu, 2004; Pink et al., 2016).

While it must be noted that I had less, if any, control over the local-level distractions in my participants’ environments and may not have even been fully aware of them (Chen and Hinton, 1999), including the small interference of push notifications from Apps on various devices, the digital nature of the communications appeared to leave my participants feeling much more relaxed than when we shared a material space.^6^ As the pre-pandemic interactions had been moderately formal in both the setting and attire as I met my participants at their work places or during a break in their work day, the discussions were also fairly serious, centering almost exclusively on my prepared questions with very minimal dialogue about personal matters. Since I had been extra vigilant of my participants’ work schedules when I was in Ukraine, the in-person meetings also only lasted approximately one hour. The fact that other people were almost always nearby, including serving staff, patrons at cafés, or other colleagues and secretaries, moreover meant that the conversations were not necessarily private. For these reasons, my interlocutors were sometimes reserved, stiff, or quiet in tone.

In direct contrast, my online participants were noticeably more comfortable. This was demonstrated by their clothing choices, which indicated a lesser degree of formality in ranging from business casual to sweat pants, with one participant in her bathrobe. Because these individuals had flexible work schedules due to the pandemic, the interviews were scheduled both during and outside of normal working hours, starting between 8:00 and 19:00 Kyiv time on both weekdays and weekends. The online conversations were also noticeably longer, evidenced by nearly 1,500 additional words in the transcripts. In many instances, my participants exposed intimate details about themselves and their everyday lives, and even more so than the in-person conversations (Jenner and Myers, 2019), including their dreams for the future and concerns regarding COVID-19 for their families, their communities, and Ukraine. I also had to initiate the end of most of these meetings as my participants were not rushed to end the calls after more than an hour—it appeared they were happy to speak about non-work and non-coronavirus issues, as well as with someone different than those with whom they were isolating. This last point was exemplified during one interview when the participant asked where I was located and then said, ‘tell me about how it is to live in London. . . it’s London!’^7^ During another conversation, the participant similarly extended the call for several minutes by asking me questions about Canada, stating that I was the first Canadian he had ever met. Importantly, my experiences counter prior literature that suggests in-person conversations are longer than those on the phone or over video call (Johnson et al., 2019); the online interviews and focus group that I conducted in the spring of 2020 were actually more detailed and conversational, and generated both a larger word count and more information than those held in Ukraine. While extra time was required for the online conversations (see also Jenner and Myers, 2019; Jowett et al., 2011), the co-present dynamic created by the Internet evidently still allowed for personable and intimate exchanges when co-location was not possible.

Due to the fairly relaxed nature of the online interactions, I often felt like I was speaking with a friend rather than a research participant—the use of the same digital platforms for socialization and research during the pandemic additionally proved difficult to ‘democratize’ interpersonal relationships in the research process (Owton and Allen-Collinson, 2014; Spears and Lea, 1994). Whereas the power dynamics of researcher–participant interactions in fieldwork are typically ‘reciprocal, asymmetrical or exploitative’ (England, 1994: 243)—as we purposefully enter the personal lives of our participants but they are less likely to enter ours (Knott, 2019)—online methods actually enabled a more symmetrical relationship with my participants. This is because they had greater agency and power in our exchanges (Fujii, 2018) by controlling my access to the field more significantly than in traditional fieldwork. As I conducted the interviews and focus group from my home in London, my participants were also able to ‘enter’ or observe my personal life in ways that would not be possible had I been in Ukraine. Though earlier works have purported that text-based and asynchronous mediated communications can encourage more equal participation by impacting the power/status differential between researchers and their participants (Kiesler and Sproull, 1992; Spears and Lea, 1994), my experiences reveal that synchronous digital approaches can also greatly implicate the power dynamics. Notably, it cannot be concluded whether the more intimate and egalitarian nature of the online interactions was due to co-presence with my participants rather than our co-location (Beaulieu, 2010); our inability to judge each other based on age, gender, and race in the same way as in-person interactions (Chen and Hinton, 1999; Spears et al., 2002); or the fact that my participants felt safer speaking to me within their own homes than in public places (Jenner and Myers, 2019). Nonetheless, the combination of a new setting and new methods for my fieldwork fundamentally changed my research experience and relationships with my participants within my ‘field sites.’

### Remote embeddedness

Although I was not able to interact with my participants in the more natural—albeit also asymmetrical—ways I otherwise would have if I had been physically located in Ukraine, I was still able to embed myself in their lives remotely. In fact, conversing about space and place with participants who were in different spaces and places than myself through the use of a digital platform—which created a new space (Ahlin and Li, 2019)—allowed for a triple layered analysis of space and place that was only possible because *of* mediated interactions. Importantly, there were no noticeable differences in the content of the conversations or the quality of the connection when using different digital platforms. Nevertheless, the participants who used Facebook and Viber typically used their phones, and thus were more mobile and able to show me more of my field sites than the participants who used Skype and Zoom on their computers. For example, one of my participants chose to do his interview over a Facebook voice call as he went for his morning walk, which provided me with a visual image of the local environment as I heard the people and animals that he passed in rural Ukraine (Chen and Hinton, 1999; Mann and Stewart, 2000). It must be noted that my computer-using interlocutors still disclosed local-level dynamics, such as the inside of their homes and their relations with the people in their lives. One participant, for instance, introduced me to his two young daughters when they entered the room he was sitting in, and near the end of the conversation, he presented his cat to the camera. Similarly, the participant who sat in his family’s backyard during the focus group introduced his mother to everyone on the call. Even though my ability to observe nuanced and unplanned happenings in the field (Fujii, 2015) was severely curtailed by the pandemic, digital methods nevertheless revealed angles of the field that would not be observed during in-person fieldwork, especially because scholars (and particularly junior, female scholars) are routinely advised to only meet in neutral spaces or places of work for reasons of safety and security.

In virtually situating myself within the less formal aspects of my participants’ lives, I was also able to see a ‘fuller picture’ of who they are. As interlocutors are typically selected for certain reasons—such as their titles, positions, and experiences—interacting with them in locations associated with these characteristics, such as their workplaces, implicitly encourages particular aspects of their identities to be expressed over others. When engaging in more casual environments, conversely, the other identities participants uphold are able to be expressed more freely, like that of parent, pet owner, and partner. While it has been suggested that mediated interactions are not rich enough to sustain meaningful social relations as researchers may miss out on non-observable phenomena (Mason, 1996), online approaches can evidently, and paradoxically, reveal insights which may otherwise be overlooked during in-person communications. Hine (2015) additionally explains that immersed online experiences encourage participants to absent themselves from other distractions and forms of engagement—such as other people in public places or work obligations—to express certain aspects of themselves they may feel unable to during in-person interactions (also Bargh et al., 2002; Spears and Lea, 1994; Spears et al., 2002). I observed this firsthand in seeing how a recording device (or lack thereof) affected my participants’ engagement with the discussion; a physical device sat in front of us during the in-person conversations while the online discussions were digitally recorded. Markedly, most online participants appeared to forget over time that the conversations were being recorded, however, those I met in-person were much more careful with their words, especially at the beginning of the conversations, and sometimes glanced at the device before speaking. Though it must be recognized that observing our participants in informal settings may mean that the particular identities we are interested in less prominent than the others they also uphold, online approaches may indeed encourage our participants to self-present a more unfettered version of their ‘true selves’ (O’Connor and Madge, 2017; Sullivan, 2012).

Furthermore, our participants’ online self-presentations might now actually be more similar to their offline self-presentations as people are becoming much more familiar with digital platforms in using them in their everyday practices (Bluteau, 2019; De Seta, 2020). Although the differences between a person’s online and offline presence must be recognized, the divide is now less stark as more of our ‘real lives’ (including both our work and social engagements) are happening virtually because of the pandemic and the availability of diverse forms of communication. By becoming more social than technological (De Seta, 2015), then, mediated methods may no longer influence data collection in the ways that earlier scholars suggested, particularly in terms of how people self-present themselves differently in-person and online (see, for example, Bargh et al., 2002; Kiesler and Sproull, 1992; Spears and Lea, 1994). Though neither face-to-face nor mediated approaches offer a total picture of the sites and people we study technology now very much allows us to embed ourselves in other contexts from a distance. In this way, knowledge produced through digital research is no longer as particular, or ‘partial,’ as it was previously, and is arguably even more valid and generalizable than even just a few months prior to the pandemic. Given this new reality, and the fact that researchers can be both co-present with, and embedded in the lives of, participants from a distance, understandings of the ‘field’ can thus no longer be limited to a geographic space with people and places ‘on’ it. Rather, the ‘field’ must be conceptualized as a continuum of spatio-temporal events and relations between people in diverse sociopolitical contexts (Ahlin and Li, 2019; Massey, 2005).

## Fieldwork following a pandemic

In challenging how we interact with the ‘field,’ my experiences with online research during the COVID-19 pandemic further push us to (re)consider the ways we understand fieldwork. Though the idea of ‘being there’ was, for a very long time, ‘the only fully publicly acknowledged model’ for fieldwork (Hannerz, 2003: 202), the reach of researchers from their own homes is now potentially global thanks to technological advancements (Markham, 2008). In blurring both international and private/personal boundaries, digital methods thence reinforce that ‘home’ and the ‘field’ are unstable categories created by the academy (Till, 2001). While we often construct emotional, spatial, and temporal boundaries between the *here* and the *there* (Till, 2001), research spaces are accordingly complex and hybrid places of dislocation involving both homes and fields. The new reality brought about by the pandemic further challenges this false dichotomy, as dividing our research and personal selves into sites of home and the field is nearly impossible when fieldwork is actually being conducted *from* our homes or offices (Hine, 2000). In this way, fieldwork may be characterized by repeatedly switching roles due to the ‘intermingling of fieldwork among other emails in [one’s] in- and out-box’ (Pink, 1999: 114), or even our regular practices of scrolling through, ‘liking,’ and sharing social media content (Bluteau, 2019; De Seta, 2020). Although some scholars purport that data collection techniques like online interviews or focus groups do not constitute fieldwork because the evidence must be gathered ‘*in context—*within the setting where the political decisions, events, and dynamics of interest took place’ (Kapiszewski et al., 2015: 14–15), digital platforms now enable the spaces where participants live much of their lives, and therefore, where much research must be initiated (Nast, 1994). As such, the assumption that immersion and engagement in field research requires co-location with our participants in a geographic space (Postill, 2017) no longer appears entirely accurate, or even realistic, in light of the COVID-19 pandemic.

Since digital technologies have allowed the field to be reachable from afar, collecting data online is thence no different from other forms of social research in that there is ‘no such thing as total immersion’ (Massey, 2003: 75); researchers have their own implicit biases and subjectively overlook things while in the field, even if unintentionally. Yet in many ways, conducting research online actually made me reflect more on my role as a researcher, and specifically, how my participants ‘saw’ me (virtually in this case) as someone intervening ‘at the social level’ (Fujii, 2016: 1150). This is because my access to my digital ‘site’ was determined by my participants, and consequently, much more weighed on my recruitment strategies and ensuring that my interlocutors attended our meetings. Although prior literature indicates that access and rapport can be difficult to establish online (Jowett et al., 2011), the development of more symmetrical ‘working relationships’ (Fujii, 2018), rather than merely rapport, proved particularly important for both accessing and uncovering the inner workings and dynamics of the places I study while at a distance. In order to build trusting working relationships with potential participants and establish researcher credibility, I efforted to show I respected their communities and them as individuals who were knowledgeable about my research topic and not merely a means to an end (i.e. as interviewees contributing to my project) (Fujii, 2018). Because my participants were easily able to investigate my personal life through social media platforms, how I presented myself on social media, including following Ukrainian pages and local groups on Facebook, was important in revealing my involvement in, and respect for, their country and communities (Bluteau, 2019; De Seta, 2020).^8^ As ‘being there’ is still very much possible through the spatial experience of the Internet (De Seta, 2015), fieldwork thus cannot simply be understood as an approach to research based on ‘personal interaction with research [participants] in their own settings’ (Wood, 2007: 123); it must also involve engagement with the spatio-temporal events and relationships occurring in both the digital and nondigital realms (Ahlin and Li, 2019; Massey, 2005).

But while mediated methods can prove useful and sometimes even reveal more details about our research sites and participants, it must be recognized that online and in-person methods do generate different types of data, and therefore, digital approaches are not necessarily appropriate for every project or type of research. One of the most obvious challenges to data collection when conducting online fieldwork is that participants require stable and regular Internet access, in addition to technological competence and platform familiarity (Deakin and Wakefield, 2014; Lobe et al., 2020). O’Connor and Madge (2017) further purport that mediated methods can be complicated to set up as they require both the researcher and their interlocutors to have the same type of appropriate software. Although this was not a major issue for my research in Ukraine, as the country is quite developed in terms of their Information Technologies sector, the ‘digital divide’ must be taken into consideration when using online approaches. Beyond the generalizing notion of ‘digital natives’ being more familiar with social media platforms, some online communication norms may also impact data collection, such as needing to have someone’s phone number to use WhatsApp and Viber, or being ‘friends’ or ‘followers’ on platforms like Facebook and Twitter. Platform design features may additionally prove problematic when accessing the ‘field,’ such as the time constraints imposed by Zoom on free subscriptions. In this way, much more preparatory work is required by researchers to find new ways to recruit, and engage with, participants in order to avoid selection biases and impacting the quality of the data. Researchers who have not yet gone to the field may also face considerable challenges in terms of access due to unfamiliarity with the local environment.

The above considerations are particularly important when conducting fieldwork in certain countries, with participants of specific socioeconomic backgrounds, and when significant rural/urban divides exist. In my case, for example, I had to accept that I would likely not be able to access some populations through online methods, specifically those living in very rural settings and in my oldest population (50 years and older), as many were not reachable through social media nor using the necessary technologies. For some individuals, techno-competence may also be inhibited by disabilities, such as dyslexia, audio and visual impairments, or other physical limitations which may make computer or phone use difficult.^9^ Furthermore, it must be acknowledged that some projects can only be conducted in-person or through full immersion as not all populations may be reachable or willing to participate due to concerns around their own personal security and privacy. For instance, some participants may not feel comfortable discussing certain topics with other people present in their own homes or for fear of coercion. Others may feel uncomfortable not knowing if anyone will overhear the conversation on the researcher’s end. In addition, it must be recognized that online methods may not be used the same way in all countries due to data protection concerns and the sensitivity of some topics, such as those involving minors, vulnerable populations, repressive states, or illicit behavior (Jenner and Myers, 2019; Sullivan, 2012). As it is likely more scholars will be using mediated methods into the future, at least temporarily, this paper therefore divulges the need for further thinking about the ways we can ensure our participants’ safety and maintain ethical integrity in online research.

## Final thoughts

Although one of the main benefits of field research is gathering firsthand experience by getting out of the ‘armchair’ and entering the sites under study, the COVID-19 pandemic has made this methodological approach incredibly difficult. In fact, the virus has, in many ways, pushed us back *into* the armchair—both in a physical and metaphorical sense—and required us to utilize new methods to conduct research from our own homes. While it may have previously been more difficult to access participants in our field sites, advancements in technology have allowed for new armchair approaches to interact with our participants, and even glimpse into their daily lives, from afar. As this paper has outlined, mediated approaches can generate valuable insight not otherwise available through the use of in-person methods which may actually be richer and more insightful, especially when discussing personal or sensitive topics (Jenner and Myers, 2019). Online fieldwork can also grant us access to audiovisual data, introduce us to new networks, and assist us in engaging with our participants and local-level dynamics in ways that would not otherwise be possible. In extending a field site in time and space beyond a specific bounded online or offline site (Hine, 2015), mediated approaches thus offer a means of observing our field sites and establishing co-presence with participants with no loss of rapport or a reduction in intimacy (Jenner and Myers, 2019). Accordingly, reaching the field no longer requires entering it in a physical sense, just as ‘returning from the field does not mean leaving the field in an absolute sense’ (Knott, 2019: 148).

As we are now in a position to think about what fieldwork might look like following the COVID-19 pandemic, questions around the suitability of online methods for social science research are thus of utmost importance. Virtual approaches proved useful long before the pandemic, especially for researchers who had restricted access to the field and as a way to increase sample size, and they will continue to be relevant with the new social—or in this case, *physical*—distancing requirements. Beyond the methodological practicalities of conducting research at the present time, though, there may also be ideological reasons for why digital methods are more favorable than in-person approaches. For instance, the use of mediated methods can encourage greater collaboration and coordination between scholars in more and less developed countries, including co-authoring and assistantships, as local expertise is another critical way for us to ‘see’ our field sites. The moral and ecological dilemmas of traveling for fieldwork as much as the pre-pandemic normal are additional concerns that will need to be deliberated going forward (Deakin and Wakefield, 2014). Further, it must be recognized that as the pandemic continues, much more of our lives, and our participants’, are being lived online, and thus, knowledge produced through physical immersion in a particular site may now be more ‘partial’ than ever before. Hence, the use of mediated methods not only challenges previously held understandings of the ‘field’ in releasing the strictures of time and place (Spears and Lea, 1994), but has inspired new questions around conducting transparent, reflexive, and ethical research. These considerations will prove imperative for the ways we understand ‘fieldwork’ within a post-pandemic world.

## Notes

1. It has been suggested that fieldwork is pursued more by female scholars (see Teele and Thelen, 2017), which points to a larger gendered implication of the pandemic within the academy.

2. This is also assuming the same research topics remain relevant during and following a global pandemic.

3. For the purpose of this paper, ‘online fieldwork’ refers to the use of the same methods as in-person ethnographic fieldwork like interviews, focus groups, and (where possible) participant observation albeit online.

4. I define ‘elites’ as those who have the ability to exert influence through ‘social networks, social capital, and strategic position within social structures’ (Harvey, 2011: 433).

5. Ukraine opened its borders for international travel following this initial lockdown on 15 June 2020.

6. Importantly, I was less aware of the distractions faced by my participants who used computers as the conversations were held over Zoom and Skype which do not pause videocalls when other Apps are opened in the way that Facebook and Viber do on phones.

7. Interview conducted on 21 May 2020.

8. I expect that many of the people I interviewed in-person prior to the pandemic also investigated me on social media; however, they were a lot busier then and it is unlikely that they were spending as much time online.

9. At the same time, some scholars have found online methods more useful when working with participants with impaired mobility and even vulnerable populations who may be uncomfortable meeting with an unknown researcher in private settings (Jenner and Myers, 2019).
